# Curricular changes: the impact on medical students knowledge of neuroanatomy

**DOI:** 10.1186/s12909-019-1907-1

**Published:** 2020-01-17

**Authors:** Mavilde Arantes, José Paulo Andrade, Joselina Barbosa, Maria Amélia Ferreira

**Affiliations:** 1 Department of Biomedicine, Unit of Anatomy, Faculty of Medicine of the University of Porto, Al. Professor Hernâni Monteiro, 4200-319 Porto, Portugal; 2 Division of Neuroradiology, Radiology Service, Portuguese Institute of Oncology, Rua Dr. António Bernardino de Almeida, 4200-072 Porto, Portugal; 3 Center for Health Technology and Services Research (CINTESIS), Faculty of Medicine of the University of Porto, Rua Dr. Plácido da Costa, 4200-450 Porto, Portugal; 4 Department of Public Health and Forensic Sciences, and Medical Education, Faculty of Medicine of the University of Porto, Al. Professor Hernâni Monteiro, 4200-319 Porto, Portugal

**Keywords:** Medicine, Students, Neuroanatomy, Teaching

## Abstract

**Background:**

Although neuroanatomy is considered an essential requirement in medical curriculum, its teaching has undergone many changes in recent years, with most medical schools starting to implement an integrated approach. The current paper describes the comparative evaluation of the neuroanatomy knowledge scores of medical students who attended two different pedagogic approaches of neuroanatomy in the Faculty of Medicine of the University of Porto.

**Methods:**

Forty fourth-year medical students who attended a traditional stand-alone approach and 42 third-year medical students who attended an integrated approach completed a written test of knowledge.

**Results:**

Although there were some significant differences, the results globally revealed no statistically significant difference between the neuroanatomy knowledge scores of the integrated and traditional education groups, with most students obtaining a passing score in both curricula.

**Conclusions:**

Our study is the first attempt to compare the knowledge acquired by medical students from two different pedagogical approaches to neuroanatomy. Although the integrated curricula were only implemented in the Faculty of Medicine of the University of Porto a few years ago, the students who attended these curricula obtained similar scores as those obtained by the students of the traditional curriculum. This finding suggests that an integrated curriculum can be, in light of curricular reform, an efficient approach to teaching neuroanatomy to medical students.

## Background

Over the past decades, we have assisted with major curriculum reform in undergraduate medical education [1]. One of the main reasons for the reform was the explosion of scientific knowledge that led to the introduction of new disciplines into medical curricula [2]. On the other hand, medical educators also started to realize that sound factual knowledge is not sufficient to produce competent physicians and that, instead, basic medical training should focus on clinical relevance [3]. To include new disciplines and concepts, medical curricula have been gradually rearranged [4]. In Europe, under the umbrella of the Bologna Process [5], most medical schools are moving towards skills-based teaching [6] that prepares students for clinical practice.

The teaching of medical anatomy, including gross anatomy and neuroanatomy, has also been affected by these educational movements [7]. Many medical schools have stopped delivering anatomy as an isolated, discipline-based course and are now teaching neuroanatomy in an integrated, clinically oriented block in considerably less time [8]. These changes, together with a need for greater clinical relevance of the basic sciences, have led to a restructuring of programme content and of the students’ learning objectives [9]. In response to these changes, new pedagogical techniques and innovative tools have also been introduced, which, in addition to allowing a faster acquisition of anatomical skills, encourage the interest, understanding, and retention of knowledge by students [10].

Based on changes in medical education, many educators have been asking themselves about the adequacy of the reformed undergraduate anatomy teaching programmes [11], with some educators demonstrating concern regarding the level of knowledge of anatomy acquired by students graduating from the new systems-based courses [12].

Although some scholars have already studied this subject, there is no clear consensus regarding the impact of integrated curricula on anatomy knowledge [13]. In their study, McKeown et al. [14] found that the knowledge of surface anatomy was lower in those medical students who undertook a system-based curriculum compared to those who undertook a traditional curriculum. McKeown et al. explained these findings by the reduction in teaching time in the system-based curriculum, resulting in the loss of surface anatomy classes. Findlater et al. [15] found that the move to supported self-directed learning resulted in an improvement in anatomy examination scores. They believe that students with supported self-directed learning are being provided with a more engaging approach to teaching than was offered previously, resulting in an improved understanding of anatomy and the retention of anatomical knowledge, while staff are able to devote time to the explanation of difficult principles and concepts. Klement et al. [16] showed higher or equivalent subject examination average scores for an integrated curriculum for first-year basic science courses, specifically for morphology, biochemistry, physiology, and neurobiology. Klement et al. explained these findings by the increase in time for independent studies, which is beneficial for student well-being and performance. In fact, a survey administered at the end of the year of the basic science courses showed that students were very pleased with the revised curriculum. There was overall student satisfaction with the new elements adopted in the content coverage and arrangement, the daily schedule, and the examination process. Specifically, 92% of the students respondents felt that the topics presented within each basic science course correlated well between courses. Another study (Cuddy et al. [17]) showed that the scores of the United States Medical Licensing Examination (USMLE) Step 1 and Step 2 were not related to the type of anatomy course offered by medical schools (traditional or integrated). Bergman et al. [18] also demonstrated no relationship between students’ knowledge of clinical anatomy and the adopted curricula.

Until now, there have been no rigorous studies in the literature about the effectiveness of these curricular changes on medical students’ knowledge of neuroanatomy, even though such research is essential to ensuring that graduating medical students have the relevant skills to practise safe medical care. Given this lack, we undertook a study to investigate the impact of curricular change on the neuroanatomy knowledge of undergraduate medical students at the Faculty of Medicine of Porto University (FMUP). The neuroanatomy course at FMUP was taught as a traditional subject until the 2013/2014 academic year. Since then, the teaching of neuroanatomy has been integrated with neurophysiology and neurohistology as a first-year subject named “Morphophysiology of the Nervous System”, following the “Morphophysiology of the Locomotor System” lecture course in the first semester.

## Methods

### Curricular background

Neuroanatomy was taught as a subject (NeuroAnat) until the 2013/2014 academic year. This subject followed the traditional teacher-centred education approach. The course had a combination of lectures (30 h) and practices and laboratory classes (45 h), consisting of the observation of prosections of the brain and spinal cord. There were 2 hours of lectures per week and 3 hours of practices and laboratory classes per week. The programme included all components of the central nervous system (CNS), as well as the organs of the senses of vision and hearing, as well as the cerebral vessels and cranial nerves. The assessment of each student included two evaluation components, a distributed exam and a final exam, both with a practical test of the identification of neuroanatomical structures and a written theoretical test with short-answer questions.

In Morphophysiology of the Nervous System (MorphoNS), neuroanatomy is delivered in a system-based approach, together with neurophysiology and neurohistology. In addition to including all components of the CNS, the programme also focuses on the organs of the senses of vision and hearing, as well as the cranial nerves, the bones of the skull and face, the temporomandibular joint, the hyoid bone, the muscles of mastication, and mimic and neck muscles. The module consists of formal lectures (a total of four lectures, 1 hour each) and theoretical-practice classes (approximately 60 h). The assessment of the students includes a final examination, with a practical component of the identification of the neuroanatomical structures and a written theoretical component with short-answer questions.

### Data collection

We planned a cross-sectional study including the third- and fourth-year medical students of FMUP. The third-year students (MorphoNS students) were the pioneers educated with the integrated curricula. The fourth-year students (NeuroAnat students) were the last cohort of students to be taught using the old traditional curriculum (Table 1).

**Table 1:** “Retention” time for the knowledge taught

Event	First Year		Second Year		Third Year		Fourth Year	
	Semester 1	Semester 2	Semester 1	Semester 2	Semester 1	Semester 2	Semester 1	Semester 2
MorphoNS		x						
NeuroAnat			x					
MorphoNS Exam						x		
NeuroAnat Exam							x	

To compare the neuroanatomy knowledge scores of the medical students enrolled in these two different pedagogic approaches of neuroanatomy (the stand-alone approach versus the integrated approach), fourth-year medical students who had completed NeuroAnat and third-year medical students who had completed MorphoNS were invited to complete a written multiple-choice examination (knowledge question examples are included as Additional file 1). The test was the same forth both groups of students and focused on essential issues relevant to clinical practice. To assess the application of neuroanatomy knowledge to clinical situations, we use patient vignettes, including patient age, gender, and chief complaint and a summary of findings.

By reviewing the educational programmes, we determined that 15 main neuroanatomy topics were common to both integrated and traditional programmes. Each topic corresponds to a domain (Domain A- spinal peripheral nerves, Domain B- spinal cord, Domain C- brain stem, Domain D- cerebellum, Domain E- basal ganglia, Domain F- subthalamus, Domain G- telencephalon, Domain H- afferent and efferent pathways, Domain I- thalamus, epithalamus, hypothalamus, hypophysis, Domain J- limbic system, Domain K- ventricular system, Domain L- meninges, Domain M- cranial nerves, Domain N- mechanisms of vision, and Domain O- mechanisms of hearing). We prepared a written test with 40 multiple-choice questions to assess the knowledge scores of students on selected subjects. The number of questions related to each topic was proportional to the time allocated for each of the topics in the curriculum. The content validity of the questions was tested by consulting experts in relevant fields. All data were collected in 2016. The scoring procedure was implemented over a total of 40 points, where each correct answer scored one point and each wrong answer scored zero points. Data were subjected to statistical analysis by a chi-square test and a t-test in SPSS 10.0. Before the application of the examination, the purpose of the study was explained to the students, and their written consent was obtained. The test was applied to third-year students (MorphoNS students) and to fourth-year students (NeuroAnat students). Neither the MorphoNS or NeuroAnat students had any neuroscience education prior to medical school. During the first 3 years of medical school, neuroscience education focuses on the teaching of neuroanatomy in the MorphoNS or NeuroAnat subjects. A rotation in neurology in the fourth or fifth year of medical school completes the neuroscience education. We excluded the fourth-year students who had completed their neurology rotation because their inclusion may have reminded them of some issues that may have been previously learned in neuroanatomy. Ethical approval for this study was obtained from the Faculty of Medicine University of Porto/São João Hospital Ethics Committee in compliance with the Helsinki Declaration.

## Results

Forty fourth-year medical students who attended NeuroAnat (the stand-alone approach) and 42 third-year medical students who attended MorphoNS (the integrated approach) were included in our study (Table 1). The mean proportion of correct responses was .64 (*M* = 25.40) for the MorphoNS students and .61 (*M* = 24.23) for the NeuroAnat students. A *t*-test for independent samples was performed to analyse the possible differences between the two groups of students. The results showed that there were no significant differences between the MorphoNS (*M* = 25.40; *SD* = 4.37) and NeuroAnat students (*M* = 24.23; *SD* = 5.15) regarding their overall scores, *t* (80) = 1.12, *p* = .265.

Table 2 shows the descriptive statistics for each domain, comparing the NeuroAnat and MorphoNS curricular unit students. The results indicate that the MorphoNS students performed significantly better than the NeuroAnat students in domain D [t (80) = 2.21, *p* = .030], domain F [t (80) = 3.53, *p* = .001], domain H [t (80) = 2.86, *p* = .005] and domain N [t (80) = 2.68, *p* = .009]. However, the NeuroAnat students had a significantly higher mean than the MorphoNS students in domain O [t (80) = − 2.53, *p* = .013].

**Table 2:** Mean proportion of correct responses (*M*) and standard deviation (*SD*), separated for the MorphoNS and NeuroAnat students

	MorphoNS students (*n* = 42)		NeuroAnat students (*n* = 40)		*P*
	*M*	*SD*	*M*	*SD*	
Domain A	.57	.24	.69	.31	*p* = .063
Domain B	.64	.40	.78	.34	*p* = .113
Domain C	.55	.34	.46	.40	*p* = .304
Domain D	.78	.16	.68	.25	*p* = .030*
Domain E	.57	.32	.69	.35	*p* = .124
Domain F	.73	.18	.58	.21	*p* = .001**
Domain G	.57	.26	.49	.23	*p* = .169
Domain H	.70	.25	.54	.23	*p* = .005**
Domain I	.62	.25	.63	.25	*p* = .826
Domain J	.86	.35	.85	.36	*p* = .928
Domain K	.80	.31	.66	.33	*p* = .060
Domain L	.31	.47	.48	.51	*p* = .129
Domain M	.71	.22	.77	.23	*p* = .281
Domain N	.56	.42	.34	.33	*p* = .009**
Domain O	.39	.36	.59	.34	*p* = .013*

* *p* < .05; ** *p* < .01

### Gender differences

Table 3 shows the mean scores for the full sample and for both males and females. A *t*-test for independent samples was performed to explore whether there were any differences between genders. No significant differences were found between males (*M* = 25.12; *SD* = 4.09) and females (*M* = 24.63; *SD* = 5.23)*, t* (80) = .46, *p* = .648.

**Table 3:** Comparison of the averages scores of males and females participants

Full Sample (*N* = 82)		Females (*n* = 48)		Males (*n* = 34)	
*M*	*SD*	*M*	*SD*	*M*	*SD*
24.83	4.77	24.63	5.23	25.12	4.09

## Discussion

In the present study, we compared a stand-alone approach with an integrated approach as a means of teaching neuroanatomy to undergraduate medical students. Both methods were compared considering the knowledge retention of the students. For this purpose, we used a multiple-choice test focused on essential issues in clinical practice.

In our study, we found that although the mean scores for the MorphoNS medical students were higher than those for the NeuroAnat medical students, this was not a statistically significant increase. However, the difference between the scores of the groups was statistically significant in five topics. The MorphoNS group scored significantly higher than the NeuroAnat group in four domains, corresponding to the “cerebellum”, “subthalamus”, “afferent and efferent pathways” and “mechanisms of vision” topics. The NeuroAnat group scored significantly higher than the MorphoNS group in only one domain, corresponding to the “mechanisms of hearing” topic. The reason for better knowledge scores among the students of the MorphoNS group in the subsections that include more complex neuroanatomical networks may result from the special educational effort to focus on functional aspects and not on excessive anatomical details. Perhaps the students in the NeuroAnat group scored better on the “mechanisms of hearing” topic because some of them had already completed a subspecialty rotation in ear, nose and throat (ENT).

Overall, the scores obtained for both approaches were quite satisfactory, with only 10% of the students failing to obtain a passing score. These results demonstrate that although the approaches are very different, both curricula are able to convey to students what is important in their future daily practice. Moreover, since the integrated approach has been implemented in FMUP for only a few years and is still being adjusted, these results are very encouraging since they suggest that had this curriculum had been installed longer, the grades might be even better. The truth is that this curriculum focuses on clinically important issues without undue detail. Overloading students with excessive anatomical details that have little or no relevance to future clinical practice demonstrates a negative perception in the teaching of anatomy [19–21].

Within the curriculum reform that is occurring around the world, there is increasingly a call for teaching medical student content that is relevant to their future clinical work or studies [22–24]. In fact, one of the goals of curriculum reform is to produce students who are capable of understanding the complex clinical problems that they will encounter as practitioners. One way of accomplishing this goal is through exemplification using clinical cases [25]. Indeed, the use of clinically relevant scenarios in teaching might also be a means to move students from technicians who are implementing procedures to scientifically educated, enquiring practitioners who are able to analyse and solve problems.

The need to incorporate clinical relevance into neuroanatomy education, associated with an overall decrease in hours, has triggered the need to redefine the programmatic content. To address these new challenges in neuroanatomy education, a core syllabus with the most clinically relevant topics was recently formulated on an international scale for the teaching of neuroanatomy to medical students [26]. In addition to helping medical educators create a very focused curriculum, identifying a core neuroanatomy syllabus that is clinically relevant may help to reduce the so-called neurophobia. The term neurophobia was introduced in 1994 by Jozefowicz [27] to describe a general perceived difficulty with the science and clinical practice of neurology. The complexity of neuroanatomy has been identified as contributing to neurophobia, which means that every effort to reduce the complexity of neuroanatomy is welcome.

The fact that only 10% of the students failed to obtain a passing score on the test allows us to infer that the loss of knowledge was low. These results are quite different from those shown in the literature. The poor retention of basic science knowledge was first described in 1965 when Sinclair found that a retest of students on lower limb anatomy given 2 years after the initial instruction gave results no better than random guessing [28]. Shulman [29] reported that the curve of forgetting information learned in gross anatomy by the end of clerkship was no different in shape than the curves representing nonsense syllables. Between 1981 and 1991, three studies were published that all found a decline in retention of basic science information after the second year of medical school, although the studies varied somewhat in design [30, 31]. Swanson et al. [32], when retesting for standardized medical licensing exams, found an invariable decline in the knowledge of basic science information on part one of the United States Medical Licensing Exam as examinees progressed through the several steps of their medical education. D’Eon [33] found a considerable knowledge loss among medical students in the three basic science courses tested, including neuroanatomy. Mateen et al. [34] found that graduating medical students did not retain enough knowledge of neuroanatomy to allow them to pass a first-year exam.

There are several limitations to our study. First, we compared groups of students who were at two different levels of their medical school training (one group representing exclusively 3rd-year medical students and the other representing 4th-year medical students). Second, the percentage of students who completed the examination was low, resulting in a weaker data set than desirable. Third, the assessment was a multiple-choice examination, which can somehow condition the respondents’ answers. Nonetheless, this study is the first attempt to compare the knowledge acquired by medical students from two different pedagogical approaches to neuroanatomy.

## Conclusions

Our study, which compares the student knowledge of neuroanatomy from integrated and traditional curricula, aims to contribute to the debate about the adequacy of curriculum reforms. Although the Bologna Process has provided us with some guidelines, it is difficult to design a neuroanatomy course because such a design is affected by the resources available in different medical schools.

Although the integrated curricula were only implemented a few years ago, the students who attended this curricula obtained similar scores as those obtained by the students of the traditional curriculum, which suggests that an integrated curriculum can be, in light of the curricular reform, an efficient approach to teaching neuroanatomy to medical students.

## Supplementary information

**Additional file 1:** Examples of multiple-choice questions based on clinical cases included in the test.

## Data Availability

The datasets used and/or analysed during the current study are available from the corresponding author upon a reasonable request.

## Abbreviations

CNS: Central nervous system
FMUP: Faculty of Medicine of Porto University
USMLE: United States Medical Licensing Examination
