# Supplementary material for: Curricular changes: the impact on medical students knowledge of neuroanatomy
Source: BMC Med Educ. 2020 Jan 17;20:20. doi: 10.1186/s12909-019-1907-1 (PMC6969452; doi:10.1186/s12909-019-1907-1)
Supplement: Supplementary file 1 — Additional file 1: Examples of multiple-choice questions based on clinical cases included in the test. [file 12909_2019_1907_MOESM1_ESM.doc]

repeated-measures

ANOVA was performed with adjustments for

multiple comparisons using the Bonferroni

tests with the signiﬁcance level set at 5%.

| A 30-year-old woman presents to her physician assistant for white fluid from her nipples. The physical examination also revealed a deficit in the visual fields. MRI revealed the presence of a macroadenoma compressing the medial portion of the optic chiasm. Based on the location of this tumor, what is the most likely visual deficit? (**Choose the CORRECT answer***).  A) Bitemporal hemianopsia  B) Right superior quadrantopsia  C) Left superior quadrantopsia  D) Right homonymous hemianopsia  E) Left homonymous hemianopsia |
| --- |
| A 67-year-old man complains to his family medicine physician that his face "is strange." The examination reveals paresthesias in the left hemiface and on the same side of the tongue. MRI shows a lesion in the cerebral cortex, on the right. This lesion is most likely located in which of the following cortical regions? (**choose the CORRECT answer**).**  A) Inferior frontal gyrus  B) Postcentral gyrus  C) Precentral gyrus  D) Lateral occipito-temporal gyrus  E) Middle temporal gyrus |
| A 27 year old woman underwent a partial thyroidectomy for a follicular adenoma. One month after surgery the patient complains to her family medicine physician of some hoarseness. What is the most likely explanation? (**choose the CORRECT answer*****).  A) Trauma of the vocal cords by the endotracheal tube.  B) Cricoid cartilage injury.  C) Thyroid cartilage injury.  D) Recurrent laryngeal nerve injury.  E) Hypoglossal nerve injury. |

**Appendix 1.** Examples of multiple-choice questions based on clinical cases included in the test.

Correct answer:

*A)

**B)

***D)
